# Mixed serous neuroendocrine neoplasm of the pancreas: Case report and literature review

**DOI:** 10.1097/MD.0000000000004205

**Published:** 2016-08-26

**Authors:** Yatong Li, Menghua Dai, Xiaoyan Chang, Wendi Hu, Jie Chen, Junchao Guo, Wenming Wu, Taiping Zhang, Quan Liao, Ziwen Liu, Ya Hu, Yupei Zhao

**Affiliations:** a Department of General Surgery; b Department of Pathology, Peking Union Medical College Hospital, Beijing; c Department of Hepatobiliary and Pancreatic Surgery, First Affiliated Hospital, Zhejiang University School of Medicine, Zhejiang, P.R. China.

**Keywords:** mixed serous neuroendocrine neoplasm, morphology, pancreas, pancreatic neuroendocrine tumor, serous cystic neoplasm

## Abstract

**Introduction:**

The aim of this study was to report a new case of mixed serous neuroendocrine neoplasm (MSNN) and review the literature concerning this type of lesion, which was added to the World Health Organization classification of pancreatic tumors in 2010.

**Results:**

A 73-year-old woman presented with a pancreatic mass. The lesion was an intriguing combination of serous cystic neoplasm (SCN) and pancreatic neuroendocrine tumor (PanNET), in which the PanNET component grew into the wall of the serous oligocystic adenoma. We searched different databases for studies that had investigated MSNN. A total of 15 patients (age, 28–78), including the patient in the present study, were evaluated. We discuss these cases in detail especially regarding morphology and pathology; our case was the only one involving a collision type combination.

**Conclusion:**

Although MSNN is recognized as a variant of SCN, it is quite different from SCN or PanNET. A new morphological analysis of MSNN may help in elucidating its histogenesis and prognosis.

## 1 Introduction

Serous cystic neoplasm (SCN) of the pancreas is a very rare benign lesion, accounting for only 1% to 2% of all pancreatic tumors.^[1,2]^ In 2010, several new types of SCN were added to the World Health Organization criteria for pancreatic tumor classification; this includes the mixed SCNs and pancreatic neuroendocrine tumors (PanNETs). These are termed a mixed serous neuroendocrine neoplasm (MSNN), and defined as a tumor containing 2 components with different pathologies.^[3]^ Several cases of MSNN have been reported, including distinctly separated and intimately admixed tumors. Here, we report a new case of MSNN, and review the previously reported cases.

## 2 Case report

**Figure 1:**
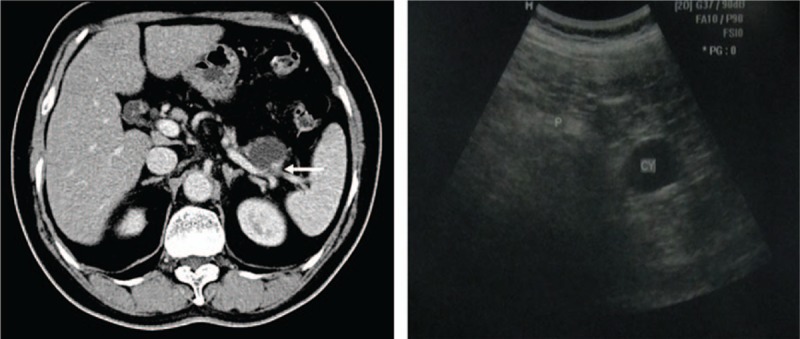
Preoperative CT and ultrasonography scans of our patient, with an ill-defined cystic lesion located in the pancreatic tail, and the arrow points the mural nodules. The tumor was 3.3 × 2.6 cm in CT scans and 3.4 × 2.5 cm in ultrasonography. CT = computed tomography.

A 73-year-old Chinese woman was admitted to our hospital in August 2010, having suffered from an incidental mild epigastric pain for 10 months. The patient had no complaints involving jaundice, vomiting, or weight loss; however, she had hypertension, and underwent an oophorotomy in 2006 because of an ovarian physiologic cyst. Examination on admission revealed impaired liver function with increased levels of ALT (133 U/L) and AST (116 U/L). The level of amylase was normal and all of the tumor markers, including CA19–9 (17.9 U/ml), CA242 (6.1 U/ml), CEA (2.46 ng/ml), AFP (15.7 ng/ml), and CA125 (10.2 U/ml), were within normal limits. A cystic tumor with a thin capsule at the pancreatic tail, which measured 3.4 × 2.5 cm in diameter, was detected using ultrasonography (Fig. 1). Enhanced computed tomography (CT) revealed an ill-defined hypovascular tumor, approximately 3.3 × 2.6 cm in diameter, located in the pancreatic tail and adhering to the splenic artery. Relatively intense mural enhancement and mural nodules were observed in the cyst (Fig. 1).

**Figure 2:**
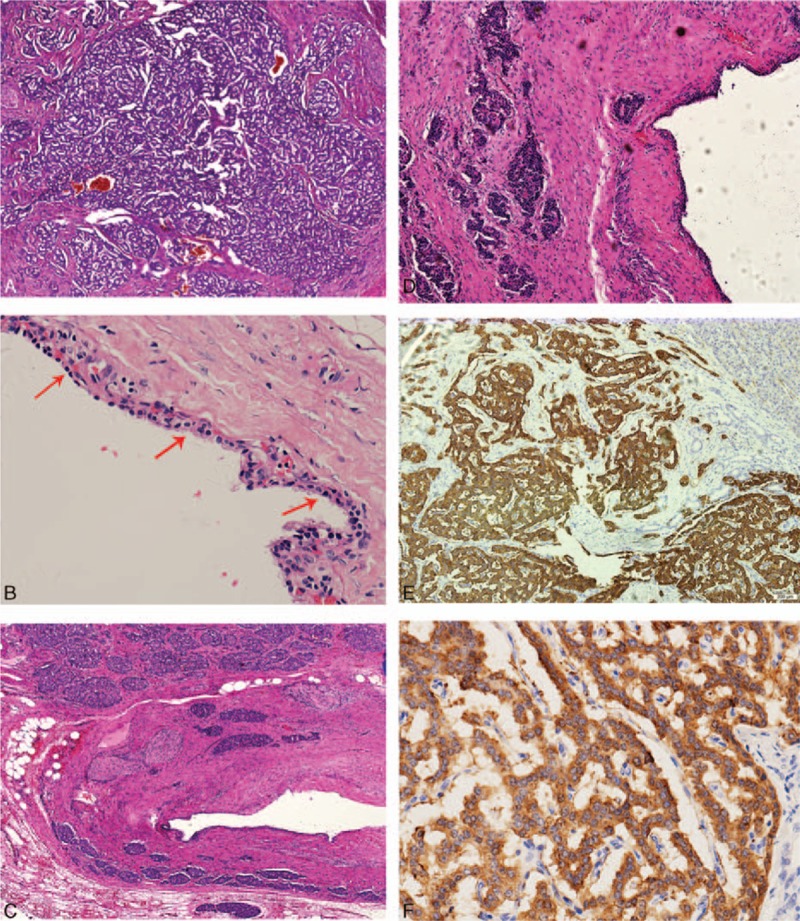
Histopathological analysis of the mixed serous neuroendocrine neoplasm (MSNN) in our case. (A) Partial endocrine component of the mixed tumor. Neoplastic cells were arranged in trabecular or banding pattern. Mitosis was scarce (150×). (B) Lining cuboidal epithelial cells (arrow) with a pale or clear cytoplasm and a centrally located round nucleus. Cytological atypia was minimal (150×). (C) The collisional growth of 2 components with the pancreatic neuroendocrine tumor (PanNET) growing into the wall of serous oligocystic adenoma. Perineural and fat tissue invasion was noted (60×). (D) High-powered magnification of the collision (150×). (E) Immunohistochemistry staining for synaptophysin. Neuroendocrine tumor cells were positive (150×). (F) Immunohistochemistry staining for chromogranin A. Neuroendocrine tumor cells were positive (300×).

The initial diagnosis was pancreatic cystic neoplasm. Laparoscopic distal pancreatectomy with splenectomy was performed, during which a 3-cm tumor was found encroaching on the pancreatic tail. The patient had an uneventful postoperative recovery and left hospital 16 days later. The resected pancreatic tissue measured 9.0 × 6.0 × 2.5 cm, together with a unilocular cystic tumor measuring 2.5 × 2.0 × 1.0 cm. The cyst was filled with serous fluid while the inner surface was white and smooth. A focal gray, solid ill-defined mass (approximately 1.2 × 1.0 × 0.5 cm in diameter) was located adjacent to the cyst. Hematoxylin and eosin staining of the surgical specimen demonstrated that the cyst was formed by a thin fibrous wall, which was lined with a single layer of cuboidal or flattened epithelial cells exhibiting partial denudation (Fig. 2B). The cell cytoplasm was pale to clear while the nucleus was round to oval and centrally located. Cytological atypia was minimal and there were no invasive or metastatic features present. According to the definition of Lee et al^[4]^ and Kimura et al,^[5]^ serous oligocystic adenoma was identified.

The adherent solid mass was composed of cells arranged in a trabecular or banding pattern. The size and shape of these cells were uniform with a low karyoplasmic ratio, and there was no mucin inside. The chromatin appeared fine and was arranged in a stippling pattern. In addition, the nucleolus was not obvious, and small nucleoli could occasionally be seen. Nuclear fission was scarce and appeared in less than 1 in every 10 high power fields of view. Perineural invasion and fat tissue permeation were noted, while the lymph nodes examined were negative. In accordance with these findings, a PanNET (G1) with uncertain behavior was considered; it was positive for the expression of chromogranin, synaptophysin, and Ki67 (index < 1%) (Fig. 2E and F). Consequently, a histopathological diagnosis of MSNN was made. Intriguingly, we found these 2 components exhibited a collision type of growth, with the PanNET component growing into the wall of the SCN (Fig. 2C and D).

The patient started to suffer from diabetes shortly after surgery. During a 54-month follow-up, there were no signs of tumor recurrence or metastasis, and the complaints of epigastric pain and abnormal liver function evident before surgery had disappeared. The patient gave her informed consent for treatment and inclusion in this study, after having been provided with all the necessary information.

## 3 Discussion

### 3.1 Review of literature

The World Health Organization classified the SCNs into 5 types in 2010^[3]^: serous microcystic adenoma, serous macrocystic/oligocystic adenoma, von Hippel–Lindau (VHL)-associated SCN, solid serous adenoma/neoplasm, and MSNN. SCNs combined with other pancreatic neoplasms have seldom been reported. To investigate MSNN, the PubMed, Embase, and Science Citation Index databases were systematically searched until February 2015. The keywords used were “serous cystic tumor/neoplasm,” “endocrine tumor/neoplasm,” “islet cell tumor/neoplasm,” and “pancreas.” The citation lists associated with all of the studies retrieved were used to identify other potentially relevant publications. The reference lists were also checked, including case and review publications. The search results were then screened according to the following inclusion criteria: any form of publications focused on MSNN, without a third pancreatic tumor; and presentation of sufficient data, including clinicopathological characteristics and histopathological features. Studies were excluded if they met the following criteria: were not published in English; and duplicated data from the same sample of patients.

The systematic search yielded a total of 14 studies comprising 17 patients.^[6–19]^ Hough et al^[7]^ reported a case of diffuse cystic changes in the pancreas associated with PanNETs, but detailed data was not available. Baek et al^[8]^ and Jung et al^[12]^ reported the same patient in Korea, while Heresbach et al^[6]^ reported a case of cystadenocarcinoma with MSNN in the pancreatic head. Thus, 14 patients remained for analysis in the current study after excluding these 3 studies.^[6–8]^ The baseline characteristics of the included studies are summarized in Table 1. As for statistical analysis, we used Fisher test, the *t*-test, and the *t*’-test when comparing different groups.

**Table 1:**
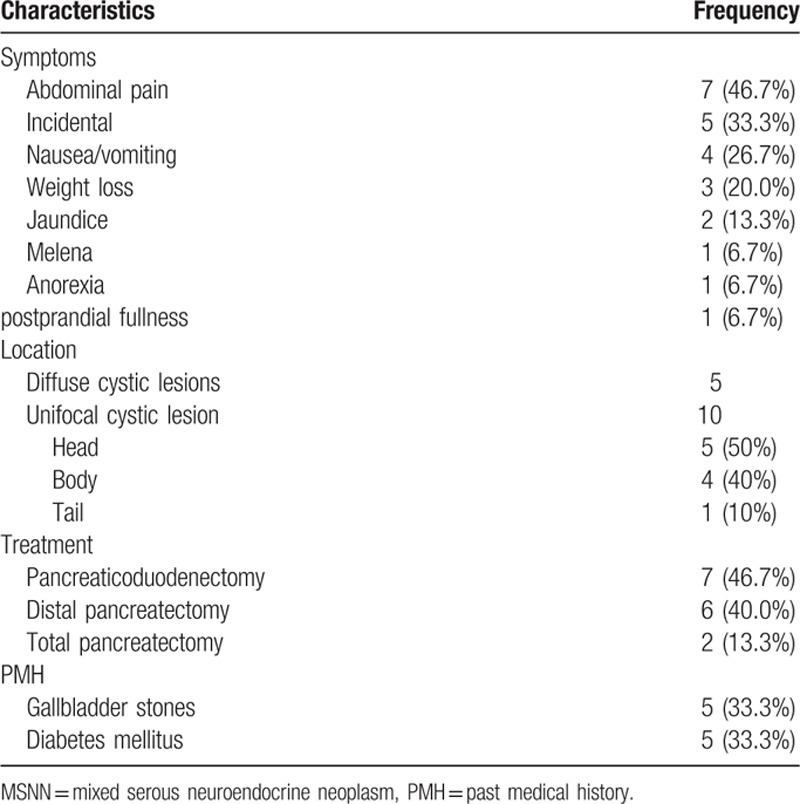
Clinical features of MSNN (15 cases).

To the best of our knowledge, our case was the 15th reported case of MSNN. After reviewing the other cases, we found that our case was the first where the mixed lesion arose in the pancreatic tail; it was also the first case with an intimately, partially overlapped but mostly separated mixed SCN and PanNET. Among these patients, 13 were female and 2 were male. The median age was 52 (range, 28–78) years. The chief complaints were abdominal pain, nausea/vomiting, weight loss, and jaundice, in 46.7%, 26.7%, 20.0%, and 13.3% of patients respectively. The symptoms could last for several years before diagnosis. There were 5 (33.3%) patients with no symptoms. The abnormalities were discovered incidentally during physical examinations. However, no endocrine syndrome was detected in any of the patients (Table 1).

The results of laboratory tests in these cases were mostly within the reference range, except for the case reported by Hsieh et al^[19]^ that involved an increased level of CA19–9. All patients underwent preoperative ultrasonography and/or enhanced thin-sliced spiral CT and/or magnetic resonance imaging scans. Multiple or diffuse cystic lesions were found in 5 (33.3%) patients. The unifocal cystic lesions were often located in the head of the pancreas (50% [10 cases]) with varying sizes, from 1.3 cm to 13 cm in greatest dimension. Most tumors were hypoechoic in ultrasonic images and exhibited low density in CT scans, with or without enhancement after contrast injection. The medical history in all 15 patients was investigated; gallbladder stones and diabetes mellitus were most frequently seen with an incidence of 5/15.

All 15 patients accepted intentional radical surgery; there were 7 pancreaticoduodenectomies, 6 distal pancreatectomies with splenectomy and 2 total pancreatectomies. Patient postoperative recovery was uneventful. The longest follow-up time was 2 years (not including our patient). None of these patients had died of tumor recurrence or distant metastasis by the time they were reported.

### 3.2 Morphological and clinicopathological analysis of MSNN

**Figure 3:**
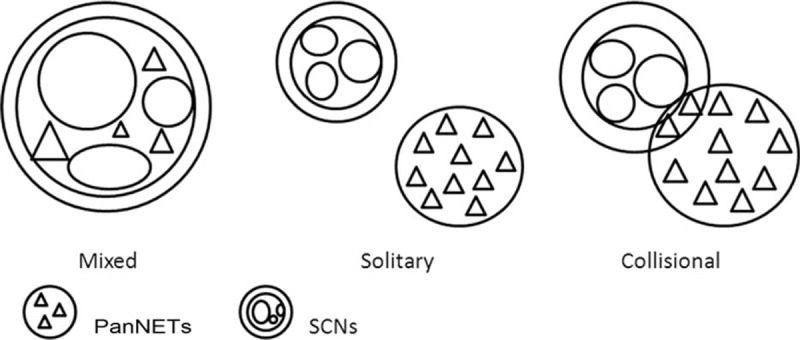
Three subtypes of mixed serous neuroendocrine neoplasm (MSNN) with different morphological features.

We analyzed all 15 cases with regard to different clinical, morphological, and histological appearances, and found that MSNN could be subdivided into several types (Fig. 3). They were as follows. The diffuse type: the entire pancreas was involved with numerous serous cysts and PanNETs, which could be located in any region of the pancreas. The mixed type: an intimate admixture of cell masses involving SCN and PanNET. The 2 components grew together and jointly constituted the tumor tissue; these components could not be distinctly divided. The solitary type: there were isolated SCNs and PanNETs arising in the pancreas without any intermingling area, and the transitional zone was normal, resembling 2 different tumors coinciding in one organ. The collision type: as in the case reported by us, most parts of these 2 components were separated with a partially intermixed or overlapping zone.

The clinicopathological analysis of these 15 patients is summarized in Table 2. There were 5 patients with diffuse SCN associated with PanNET, 5 with mixed ones, and 4 with solitary ones. We noted that the diffuse type was mostly associated with VHL disease (3 confirmed, 1 suspected, and 1 uncertain), and that all of the PanNET components were well-differentiated endocrine carcinomas. All the mixed type of MSNN presented as serous microcystic adenoma with larger cysts as compared with those of the solitary type (mean 9.1:1.9 cm; *P* < 0.05). Serous microcystic adenoma or serous oligocystic adenoma could both be discovered in the solitary type, in which all of the PanNETs were benign. There were no significant differences between the mixed and solitary types regarding patient age or the location of the mass.

**Table 2:**
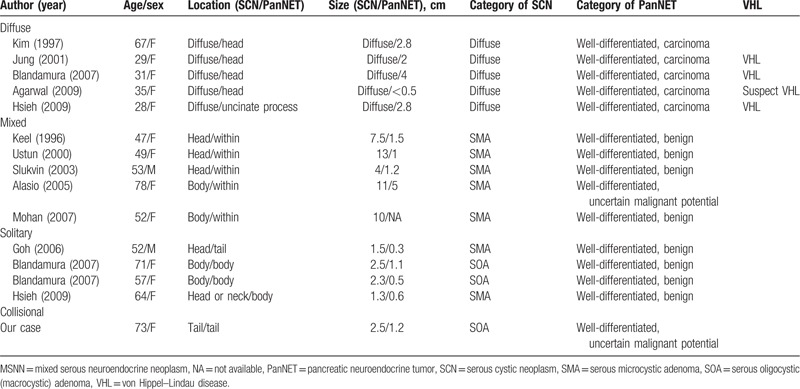
Summary of MSNN in all cases.

Our case was an unequivocal type of coexistent SCN and PanNET which was intimately adjacent to a partially intermingling area, and was termed a collision type combination (Fig. 3). Only one case of the collision type has been reported, and it seemed to have the same clinical and histological features as mixed MSNN. Thus, further investigation is needed.

### 3.3 Surgical indication and prognosis of MSNN

Because of the coexistence of 2 types of tumor, we should consider them together when discussing the surgical indication and prognosis. Generally, SCNs are benign and periodic observation is advised.^[1]^ Nevertheless, there is a challenge in accurately distinguishing SCN from mucinous cystic neoplasms, which are highly suspected of being malignant. Therefore, there are conflicting recommendations concerning the management of SCNs. PanNETs are usually regarded as being potentially malignant because their behavior is difficult to predict from their histopathological features and cellular products. Surgery is considered to be the first-line therapy for patients with PanNETs when the lesion is resectable.^[20,21]^

Generally speaking, the prognosis of MSNN depends on the malignant component. As suggested by Slukvin et al^[13]^, MSNN may have a higher malignant potential than SCN alone. Among these patients, including our case, malignant biological behavior such as perineural, lymphatic, and duodenal invasion appeared in 33.3% (5/15) of all the cases, which is much higher than that of SCN or PanNET alone. As a result, once a diagnosis of MSNN is made, we are more inclined to surgical treatment; in the cases that have been followed up, this lesion seemed to have a better prognosis after curative surgery.^[9,11,15,16,18,19]^ However, the robustness of our treatment recommendation is diminished by the small number of cases. Evaluation of the malignant potential and prognostic outcomes of mixed tumors requires a long-term follow-up and data from additional reports of such cases.

If MSNN is associated with VHL disease, it requires further consideration. High potential for malignancy has been reported in VHL-associated PanNETs.^[22,23]^ Among the patients, including our case, 3 VHL-SCNs were described; all these cases had diffuse lesions and invasion. Despite the fact that no metastasis was seen, these 3 cases should be attributed to neuroendocrine carcinoma. Therefore, close attention should be paid to the presentation of diffuse MSNN regarding VHL disease. Surgical treatment is suggested and the prognosis may not be so optimistic. Close clinical follow-up and aggressive resection are recommended.

## 4 Conclusion

MSNN is a distinct clinicopathological entity rather than an incidental concurrence of 2 separate tumors. To the best of our knowledge, we are the first to report a collisional case of MSNN, followed by a literature review and analysis based on its morphological and clinicopathological appearance. Different types of MSNN exhibit various features that could be helpful in indicating the prognosis. However, considering the limited number of cases reported, further study is necessary.
